# Attenuated *Leishmania major* Induce a High Level of Protection against *Leishmania infantum* in BALB/c Mice

**Published:** 2019

**Authors:** Shamsi NOORPISHEH GHADIMI, Leila HOMAYOON, Reza SHAHRIARIRAD, Shakila FATEHPOUR, Mohammad RASTEGARIAN, Bahador SARKARI

**Affiliations:** 1. Department of Parasitology and Mycology, School of Medicine, Shiraz University of Medical Sciences, Shiraz, Iran; 2. Student Research Committee, Shiraz University of Medical Sciences, Shiraz, Iran; 3. Basic Sciences in Infectious Diseases Research Center, Shiraz University of Medical Sciences, Shiraz, Iran

**Keywords:** Attenuated *L. major*, *L. infantum*, Cross-protection

## Abstract

**Background:**

The current study aimed to investigate the possible cross-protective effects of attenuated *L. major* against *L. infantum* in BALB/c mice.

**Methods:**

This experimental study was performed in 2017 in Shiraz University of Medical Sciences, Shiraz, Iran. The attenuated strain of *L. major* was prepared by continuous weekly subculturing of the parasite. Forty-eight female BALB/c mice were divided into eight groups. Group 1 injected (ID) with wild type of *L. major*; group 2 injected (IV) with *L. infantum*; group 3 injected (ID) with attenuated *L. major*; group 4 injected (ID) with attenuated *L. major*, and after three weeks challenged (IV) with *L. infantum*; group 5 injected (IP) with attenuated *L. major*; group 6 injected (IP) with attenuated *L. major*, and challenged (IV) with *L. infantum* (IV); group 7 injected (IV) with attenuated *L. major*; and finally group 8 injected (IV) with attenuated *L. major* and after three weeks challenged (IV) with *L. infantum*. Forty-five days post-infection, the parasite load in the spleen and liver of the mice was determined as Leishman-Donovan units (LDU).

**Results:**

The differences in mean of LDU of spleen between different groups were statistically significant (*P*<0.048). In addition, the differences in percent of infection in liver between pairwise comparisons of groups were statistically significant (*P<*0.05). The highest intensity of infection was observed in group 2 while low intensity of infection was seen in groups 3, 4 and 5.

**Conclusion:**

Live attenuated *L. major* can induce substantial protection against *L. infantum,* particularly when the parasites were injected intravenously.

## Introduction

Leishmaniasis is an important vector-borne disease caused by protozoan parasites belongs to the *Leishmania* species. The disease has a wide range of clinical symptoms, from the self-healing cutaneous lesions to the fatal visceral form, based on the host’s immune responses and the *Leishmania* parasite strain involved (1). About 12 million people are presently infected with different forms of leishmaniasis, and the cutaneous leishmaniasis is responsible for most of the cases.

Although some progress has been made in controlling leishmaniasis, it is still one of the most important health challenges in some of the tropical and subtropical countries in the world (2, 3). Control of leishmaniasis involves attempts to treat clinical cases, and by developing an effective vaccine (4).

So far, a large number of vaccines, based on killed promastigotes, recombinant proteins or sequenced genes encoding the proteins of *Leishmania* parasite have been developed and evaluated in murine models, canine or human. Despite the enormous studies done on the vaccine development against CL and VL, so far no effective vaccine against human leishmaniasis is available. Satisfactory results have obtained with those vaccines developed to prevent canine leishmaniasis, which contributes to the control of reservoirs of visceral leishmaniasis (5, 6).

In Iran, the Mediterranean form of visceral leishmaniasis (VL) is common caused by *L. infantum* and it is endemic in different parts of the country, particularly in Fars and East Azerbaijan Provinces (2, 3, 7–11).

One of the subjects attracted the attention of researchers is the possibility of cross-protection induced by using strains of *L. major* to provide immunity against VL and vice versa. Study on cross-immunity between *Leishmania* species may be a useful approach to find the most appropriate parasites and the related antigen for protection against different forms of the disease.

Immunization of mice and hamsters with live attenuated certain gene-deleted *L. donovani* induces immunity against *L. donovani* and cross-protected the mice against infection with *L. braziliensis* (12). Immunization of mice with *L. infantum* sterol 24-c-methyltransferase SMT formulated with monophosphoryl lipid A in stable emulsion (MPL-SE) induce cross-immunity against *L. major* (13). Experimental infection with *L. major* in monkeys has induced significant protection against *L. amazonensis* and *L. guyanensis*, but not against *L. braziliensis* (14).

We aimed to investigate the possible cross-protective effects of attenuated *L. major* against *L. infantum* in BALB/c mice.

## Materials and Methods

### Leishmania parasites

This experimental study was performed in 2017 in Shiraz University of Medical Sciences, Shiraz, Iran. *L. major* (MRHO/IR/75/ER) and *L. infantum* (MCAN/IR/07/Moheb-gh), which were available in the Department of Parasitology and Mycology of the School of Medicine at Shiraz University of Medical Sciences, Shiraz, Iran, were used in this study. Amastigotes of *L. major* were obtained from the lesion of infected BALB/c mice and transferred into NNN medium. The parasite then mass cultivated in RPMI medium (Gibco), supplemented with 10% inactivated fetal calf serum (SBFI), 100 μg/mL streptomycin (Sigma-Aldrich, St. Louis, USA) and 100 U/mL of penicillin.

### Preparation of attenuated L. major

The attenuated strain of *L. major* was prepared by continuous weekly sub passages (21 times) of the parasite in PRMI 1640 medium (15).

### Infecting of BALB/c mice with Leishmania parasites

The animal care and the experimental protocols were approved by the ethical committee of Shiraz University of Medical Science (SUMS). Female BALB/c mice (4–6 wk old) were provided by Pasteur Institute of Iran. Mice were maintained in specific-pathogen-free conditions. The animals were divided into eight groups. The first group (control group) was inoculated, intradermally, with the wild type of *L. major* (0.2 mL injection, containing 3× 10^8 promastigotes) at the tail base. The second group was intravenously inoculated with *L. infantum* (100 μL injection, having 10^8 promastigotes). The third group intradermally received attenuated strain of *L. major* (0.2 mL injection, containing 3×10^8 promastigotes). The fourth group received attenuated strain of *L. major* intradermally. After three weeks was intravenously infected with *L. infantum.* The fifth group was infected with an attenuated strain of *L. major,* intraperitoneally (0.2 mL injection, containing 3×10^8 promastigotes). The sixth group received attenuated strain of *L. major,* intraperitoneally and subsequent (after 3 wk) injection with *L. infantum.* The seventh group was intravenously injected with the attenuated strain of *L. major* (100 μL injection, having 10^8 promastigotes). Finally, the eighth group received attenuated strain of *L. major,* intravenously. They were infected after 3 wk, with *L. infantum.*

At the end of the experiments (45 days post-infection), the mice were euthanized. Livers and spleens were removed for evaluation of the parasite burden. Touch smears were prepared from each tissue and Giemsa stained for assessment of parasite load. The parasite burden in the liver and spleen of the mice was determined as Leishman–Donovan units (LDU). The LDU index defined by using the formula LDU = amastigote number per 1,000 host cell nuclei × organ weight (in grams) (16). Moreover, the ratio of the cells containing amastigote, to the uninfected cells and intensity of infection (mean of parasite number in infected cells) were determined (17).

### Statistical analysis

The findings of the experiments were recorded in a predesigned data sheet. Data were entered into SPSS for Windows version 18 (SPSS Inc. Chicago. LL, USA). Kruskal–Wallis test (non-parametric independent group comparisons) was used to compare the mean values of the LDU between each group. Mann-Whitney test was used to compare the data in pair groups.

## Results

Mean of the LDU ranging from 0.00 (group 5) to 7387.50 (group 2) in the spleen, and from 956.66 (group 7) to 24166.66 (group 2) in the liver. The percent of infection in the spleen was ranging from 0.00 (group 5) to 5.68 (group 2) and the rate of intensity of infection in the spleen ranged from 0.00 (group 5) to 0.83 (group 1). Meanwhile, the percent of infection in liver was ranging from 0.91% (group 5) to 13.9 % (group 2), whereas the rate of intensity of infection was from 0.33 (groups 3, 4 and 5) to 1.58 (group 2). Table 1 shows the mean of LDU in different groups of mice in this study.

**Table 1:** Mean of LDU, mean of percent, and mean of the intensity of infection in liver and spleen in different groups of mice

***Different mice groups***	***Mean ± Std. of LDU in the liver***	***Mean ± Std. of LDU in the spleen***	***Mean ± Std. of percent of infection in the liver***	***Mean ± Std. of intensity of infection in the liver***
Group 1: Received wild-type *L. major*	14384.83 ± 7278.99	4302.50 ± 3510.62	3.7033 ± 2.09057	0.8500 ± .41833
Group 2: Inoculated (IV) with *L. infantum*	24166.66 ± 2309.68	7387.50 ± 7365.18	13.9500 ± 4.29127	1.5833 ±.49160
Group 3: Infected (Id) with the attenuated strain of *L. major*	10219.16 ± 23134.94	2957.50 ± 6252.68	1.3500 ± 2.29412	0.3333 ± .51640
Group 4: Infected (Id) with the attenuated strain of *L. major* and challenged (IV) with *L. infantum*	1227.00 ± 1926.05	817.50 ± 1349.62	1.7167 ± 2.98692	0.3333 ± .51640
Group 5: Infected (IP) with the attenuated strain of *L. major*	1505.00 ± 3456.66	0.00 ± 0.00	0.9167 ± 1.69046	0.3333 ± .51640
Group 6: Infected (IP) with the attenuated strain of *L. major* and challenged (IV) with *L. infantum*	3140.00 ± 2205.44	1551.10 ± 1945.34	3.8000 ± 2.65707	0.6667 ± 1.21106
Group 7: Inoculated (IV) with the attenuated strain of *L. major*	956.66 ± 1225.99	746.65 ± 968.77	1.3633 ± 2.06385	0.5000 ± .54772
Group 8: Inoculated (IV) with the attenuated strain of *L. major* and challenged (IV) with *L. infantum*	4438.00 ± 1957.01	2083.10 ± 1200.64	4.4500 ± 2.28976	0.9833 ± 04082

### Reduction of parasite load in the spleen of mice vaccinated with attenuated L. major after challenging with L. infantum

The highest mean of parasite burden in the spleen was seen in group 2 of the mice. As expected, intravenous injection of *L. infantum* caused the dissemination of the parasite in the spleen, whereas the lowest mean of parasite burden in the spleen was seen in group 5 which intraperitoneally received live attenuated *L. major*.

**Fig. 1:**
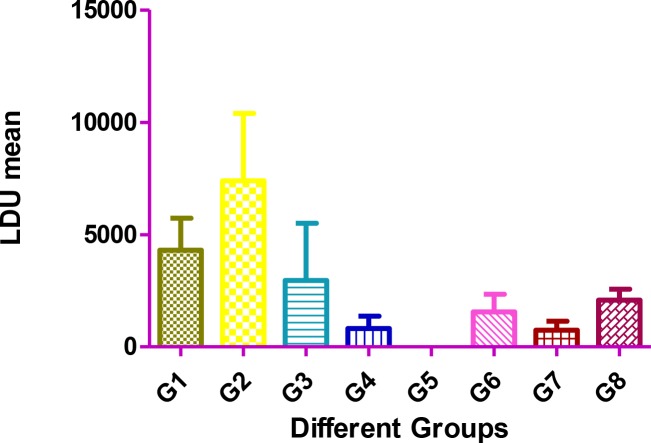
Mean of parasite burden in spleen in different groups of mice. At 45 days after immunization, spleens were removed and LDU were measured. Group 1: injected (ID) with wild type of *L. major*; Group 2: injected (IV) with *L. infantum*; Group 3: injected (ID) with attenuated *L. major*; Group 4: injected (ID) with attenuated *L. major*, and challenging (IV) with *L. infantum*; Group 5: injected (IP) with attenuated *L. major*; Group 6: injected (IP) with attenuated *L. major*, and challenged (IV) with *L. infantum* (IV); Group 7: injected (IV) with attenuated *L. major*; Group 8: injected (IV) with attenuated *L. major* and challenged (IV) with *L. infantum.*

The differences in parasite burden of spleen between the control groups and P21 attenuated *L. major*-immunized mice were statistically significant *(P<*0.048*).* Fig. 1 shows the mean of LDU of the spleen in different groups of mice. Mice of group 5, which intraperitoneally received the attenuated strain of *L. major* had the lowest mean of LDU.

### Reduction of liver parasite burden of mice vaccinated with attenuated L. major after challenging with L. infantum

**Fig. 2:**
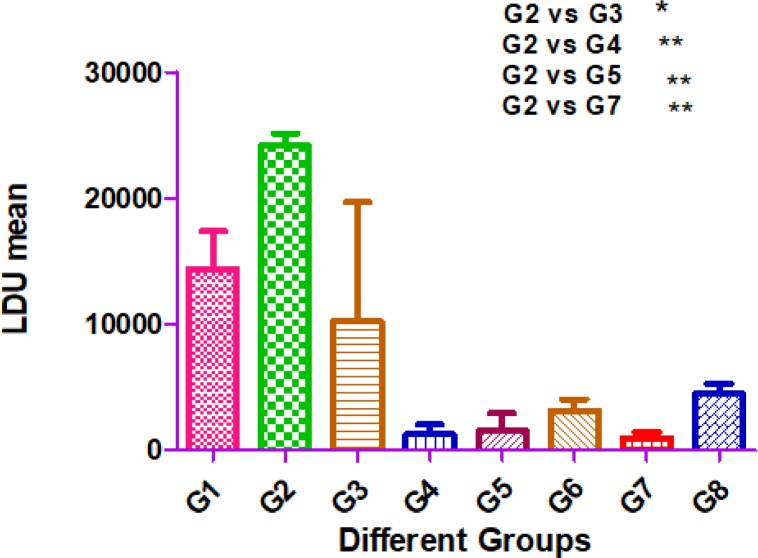
Liver parasite burden in different groups of mice. Group 1: injected (ID) with wild type of *L. major*; Group 2: injected (IV) with *L. infantum*; Group 3: injected (ID) with attenuated *L. major*; Group 4: injected (ID) with attenuated *L. major*, and challenging (IV) with *L. infantum*; Group 5: injected (IP) with attenuated *L. major*; Group 6: injected (IP) with attenuated *L. major*, and challenged (IV) with *L. infantum* (IV); Group 7: injected (IV) with attenuated *L. major*; Group 8: injected (IV) with attenuated *L. major* and challenged (IV) with *L. infantum.* * Degree of significant value (*P* < 0.05)

The differences of parasite burden in the liver between pairwise comparisons of groups were statistically significant (*P*<0.01*).* Parasite burden of the liver in the control groups and P21 attenuated *L. major*-immunized mice was different and the difference was statistically significant (*P* <0.05*).* Mice of group 1, which received wild-type *L. major* and group 2, which intravenously received *L. infantum,* presented a high liver parasite burden. As expected, control mice that received wild type of *L. major* developed a high parasite load. Mice of group 2, which were intravenously inoculated with *L. infantum*, presented the highest liver and spleen parasite burden. On the other hand, mice of group 7, with intravenous injection of attenuated *L. major,* had the lowest mean of liver LDU. Finally, the highest level of parasite burden was seen in mice of group 2, which intravenously received *L. infantum.* Fig. 2 shows the mean of parasite burden in different groups of mice.

**Fig. 3:**
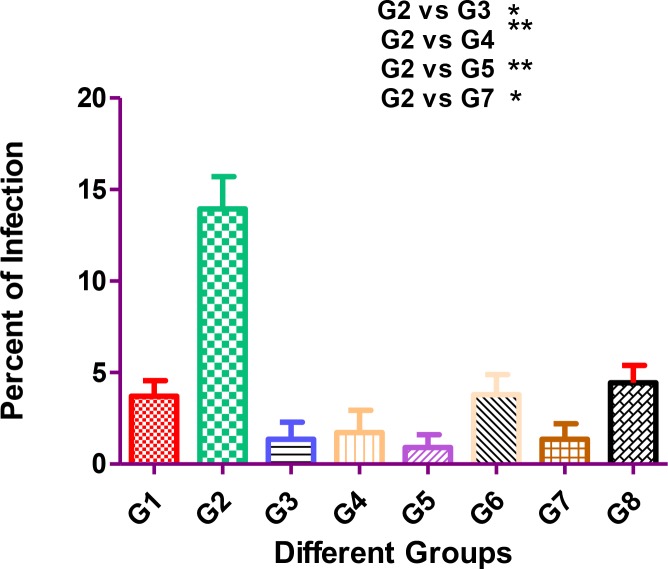
Percent of *Leishmania* infection in the liver of different groups of mice. Group 1: injected (ID) with wild type of *L. major*; Group 2: injected (IV) with *L. infantum*; Group 3: injected (ID) with attenuated *L. major*; Group 4: injected (ID) with attenuated *L. major*, and challenging (IV) with *L. infantum*; Group 5: injected (IP) with attenuated *L. major*; Group 6: injected (IP) with attenuated *L. major*, and challenged (IV) with *L. infantum* (IV); Group 7: injected (IV) with attenuated *L. major*; Group 8: injected (IV) with attenuated *L. major* and challenged (IV) with *L. infantum*. *Degree of significant value (*P*<0.05)

The differences in percent of infection in liver between pairwise comparisons of groups were statistically significant *(P*<0.05*).* The percent of infection in liver between the control group and P21 attenuated *L. major* - immunized mice were statistically significant *(P* <0.001*)*. Fig. 3 shows the percent of infection in the liver in different groups of mice. In addition, the percent of infection in liver was ranging from 0.91% (group 5: the lowest level) to 13.9% (group 2: the highest level), whereas the rate of intensity of infection was from 0.33 (groups 3, 4 and 5: the lowest level) to 1.58 (group 2: the highest level).

**Fig. 4:**
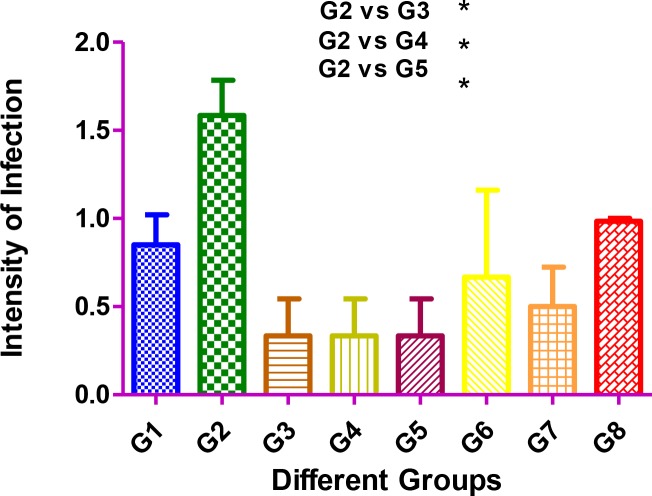
Intensity of infection in the liver in different groups of mice. Group 1: injected (ID) with wild type of *L. major*; Group 2: injected (IV) with *L. infantum*; Group 3: injected (ID) with attenuated *L. major*; Group 4: injected (ID) with attenuated *L. major*, and challenging (IV) with *L. infantum*; Group 5: injected (IP) with attenuated *L. major*; Group 6: injected (IP) with attenuated *L. major*, and challenged (IV) with *L. infantum* (IV); Group 7: injected (IV) with attenuated *L. major*; Group 8: injected (IV) with attenuated *L. major* and challenged (IV) with *L. infantum*. *Degree of significant value (*P*<0.05)

The differences in the intensity of infection in liver between pairwise comparisons of the group were statistically significant *(P*<0.05). The highest level of infection was observed in group 2 and the lowest intensity was seen in groups of 3, 4 and 5. Fig. 4 shows the intensity of infection in the liver of different groups of mice.

## Discussion

The study of cross-protective immunity among *Leishmania* species has a great application since vaccination with one species may induce protective responses against the other *Leishmania* species. In the current study, mice were immunized with live attenuated *L. major* and challenged, after 45 days, with *L. infantum*. By doing this, we determined the protective effect of the live attenuated (P21) *L. major* against the *L. infantum* infection.

The study demonstrated the reduction of parasite burden in spleen and liver of mice, which received attenuated *L. major* before challenging with wild type of *L. infantum.*

Cross-immunity among different species of *Leishmania* varies and depends on factors such as the time between recovering from the first infection and the next challenge. Conserved immunodominant antigens between different species are another key factor in inducing a cross-protection between different species of *Leishmania*.

Highly conserved antigens between different species of *Leishmania* would be a suitable candidate for inducing cross-immunity between *Leishmania* species. Among these conserved antigens, *Leishmania* large RAB GTPases proteins (LmlRAB) is highly conserved among *Leishmania* species and strains (18). Moreover, the NH36 is an important phylogenetic marker, which is highly conserved in *Leishmania* species and is a strong candidate to develop the bivalent cross-protective vaccine against both cutaneous and visceral leishmaniasis (19, 20). LEISH-F1 has entered the phase II clinical trial (21). Both LmlRAB and LEISH-F1 proteins were able to induce significant amounts of IFNγ cytokine, a key cytokine that has the main role in eliminating of *Leishmania* parasites (22).

Findings of our study demonstrated that route of administration of attenuated *L. major* is important in inducing a cross-protectively against *Leishmania* species. This is in agreement with previous studies where irradiated *L. donovani* promastigotes induced protection against *L. major* in BALB/c mice when intravenously injected (23).

The cross-protection of *Leishmania* species was found in animals infected with *L. donovani* subclinically, which developed immunity against *L .major*. Four of the five animals infected with *L. donovani* did not develop *L. major* lesion, whereas one of the animals developed a transient lesion. High levels of IFNγ were secreted in peripheral blood leukocyte (PBL) from the five animals when stimulated with *L. major* antigen. Moreover, the antibody titers indicated a high level of cross-reactivity (24).

A study on rhesus monkeys (*Macaca mulatta*) in Brazil showed that experimental infection of monkeys having self-limiting cutaneous leishmaniasis with *L. major* induced significant protection against *L. braziliensis* and *L. guyanensis*. Monkeys cured of *L. braziliensis* (cutaneous form) or *L. chagasi* (visceral form) were also immunized against *L. braziliensis* and L. *amazonensis* (14). *L. major* induces immunity against *L. amazonensis* and *L. guyanensis* in rhesus macaques while recovering from *L. major* and *L. amazonensis* did not provide protection against *L. braziliensis* infection (14).

## Conclusion

Attenuated *L. major,* as a vaccine candidate, can induce a high level of protection against *L. infantum.* Although there are differences in the immune responses against different *Leishmania* species, yet the development of a bivalent vaccine against leishmaniasis is achievable. In light of these observations, it will be possible in the future to identify the mechanism, which is responsible for the induced cross-protection of *L. major* against *L. infantum* infection.
